# A Reliability Scheduling Algorithm for the Static Segment of FlexRay on Vehicle Networks †

**DOI:** 10.3390/s18113783

**Published:** 2018-11-05

**Authors:** Trong-Yen Lee, I-An Lin, Jun-Jie Wang, Ju-Tse Tsai

**Affiliations:** Department of Electronic Engineering, National Taipei University of Technology, Taipei 10608, Taiwan; cabade7167@gmail.com (I.-A.L.); apple131212@gmail.com (J.-J.W.); abc129504323@gmail.com (J.-T.T.)

**Keywords:** FlexRay, transient fault, scheduling, re-transmission mechanism, communication reliability, time-triggered

## Abstract

FlexRay is a next-generation in-vehicle communication protocol which works in real time with flexibility. The most common applications in FlexRay are high bandwidth. X-by-wire applications, such as brake by wire and throttle by wire. However, there is no mechanism which can prevent transient faults in the application layer of FlexRay. If a transient fault occurs during driving, this would be very dangerous; therefore, we propose a fast reliability scheduling algorithm (FRSA) to improve the communication reliability of FlexRay. The proposed method reduces the probability of transient faults in one clock cycle by using a retransmission mechanism to recover the transient errors, and further improves computational complexity using the lookup table method to ensure system reliability. In this paper, we analyze a related literature to establish the system reliability constraints needed to evaluate the necessary time and slot usage, and the proposed cost function is used to evaluate the performance and efficiency when the number of messages is increased. Experimental results show that the proposed FRSA reduces execution time by an average 70.76% and cost by an average 13.33% more than the other existing methods. This method can be useful to others, especially regarding research about periodic time-triggered communication systems.

## 1. Introduction

Next-generation automotive systems will be equipped with an increasing number of components, such as electronic control units (ECUs) and sensors, which are connected through various buses. In order to deal with the amount of data and signals between ECUs or sensor nodes, traditional point-to-point communication has been replaced by vehicle networks such as controller area network (CAN) and FlexRay [1]. Modern automotive systems are moving from CAN to FlexRay. FlexRay is an automotive networking standard, and it has been increasingly adopted in the vehicle dynamics domain and inter-domain communications [2]. The advantages of FlexRay are its real-time operation, flexibility, a maximum data rate of up to 10 Mb/s, two communication channels, precise control and fault tolerance when compared with other vehicle networks. These characteristics mean that FlexRay is a fast and dependable network protocol and is often used for new safety features, such as X-by-wire [3] applications, where deterministic performance is critical. In a FlexRay bus, the communication cycle level consists of static segments, dynamic segments, symbol windows, and network idle time. Static segments were designed using a time-division multiple-access (TDMA) mechanism, and are accessed by time-triggering for safety-critical systems such as drive-by-wire, adaptive cruise control (ACC), and antilock braking systems (ABS) [4,5]. However, radiation, crosstalk, electromagnetic interference (EMI) and power supply noise in the environment cause a lot of interference for the vehicle network [6]. If a transient fault occurs, it will result in transmission delay, data loss, and a miscalculation in logic, which is very dangerous during driving. However, FlexRay does not have an application layer scheme to avoid transient faults in the static segment. The time slot of the static segment is used to store messages which should be transmitted periodically. 

Therefore, many studies have been performed on how to offer increased reliability for FlexRay. First, Li et al. [7] formulated the scheduling problem as a mixed integer linear program. This method improves scheduling by minimizing the latencies of the acknowledgement and re-transmission messages, but this work offers no guarantees for reliability. In contrast to [7], Tanasa et al. [8] proposed an algorithm which provides formal guarantees that the generated fault-tolerant message is scheduled in the static segment of FlexRay. However, this algorithm cannot generate the optimal retransmission scheme to reduce the slot utilization. In [9], Li et al. proposed a heuristic algorithm H-I method. They designed an evaluation function to determine which message should be re-transmitted, and its performance is better for bandwidth utilization and execution time than Tanasa’s method [8]. The work in [10] by Lee et al. proposed a method to ensure the reliability of systems using a reliability three-step (RTS) method. This method cannot satisfy the system reliability goal when the number of messages exceeds 25 because the slot utilization has reached 100%; this means that there is an insufficient number of slots to use in the testing time (*τ*). Wang et al. [11] proposed a frame-packing algorithm based on transmission reliability (FPBTR) to optimize signal combinations and static slot allocations. However, the FPBTR method will have a greater time cost for meeting the demands of the desired reliability. Liu et al. [12] proposed a prompt retransmission mechanism (PRTM) method to ensure the safety-critical communications of FlexRay; they pointed out that retransmission should be studied to optimize the bandwidth utilization. For these reasons, we proposed a new fast reliability scheduling algorithm (FRSA) by considering the low complexity and optimized static slot allocations in [13]. In this paper, we describe the proposed FRSA in more detail to improve the reliability in the static segment of FlexRay communication, which increases the reliability of messages by re-transmission. In addition, the proposed FRSA makes a significant improvement in running time by using lookup table method compared to the previous algorithms, even in the case of a greater number of messages; this will be shown in the experimental results.

## 2. Proposed Methodology

This section presents the proposed fast reliability scheduling algorithm (FRSA) and its system architecture, including the re-transmission mechanism to recover transient errors, how to implement the static segment of FlexRay, and the communication reliability improvement method.

### 2.1. System Architecture and Design

**Figure 1:**
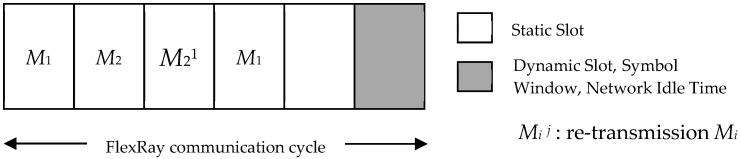
Diagram of message re-transmission mechanism.

The retransmission mechanism to recover transient errors is popularly used in communication [12,14]. The proposed retransmission mechanism generates a fault-tolerant scheduling framework by transmitting the same message repeatedly, as shown in Figure 1. 

The retransmission mechanism (i.e., duplicating messages) is based on time redundancy mechanisms in the static segment of FlexRay. In the example, we assume that the probability of failure (*PF_i_*) of *M*_2_ is bigger than *M*_1_. When the process occurs without a message re-transmission mechanism, the values of the re-transmission times (*RT_i_*) of *M*_1_ and *M*_2_ are both 0. For a system with a message re-transmission mechanism, *M*_2_ should be retransmitted because *M*_2_ has a lower reliability than *M*_1_. We now modify *RT*_1_ and *RT*_2_ to be 0 and 1, respectively.

The message will be successfully delivered with the retransmission mechanism to avoid transient failure. The reliability of each message (*M_i_*) is closely related with the retransmission times (*RT_i_*) and probability of failure (*PF_i_*). Message reliability is estimated according to Equation (1), and is equal to that shown in [8]. According to the global success reliability (*GS*), obtained by multiplying the probability of each message, increasing the probability of the successful delivery of each message will help to further improve the reliability of the system. In the fault-tolerant scheduling framework, *M_i_^j^* means the retransmitted message, where *i =* 1, 2, …, *n*, and *j* are the retransmission times to identify whether a message type is a copy or new, and its range is 1 to the total number of slots in the static segment to ensure that there are enough slots for retransmission:

(1) $$\;{{(1\;-\;PF}_{i}^{{\;RT}_{i}+1})}^{\frac{\tau }{T_{i}}}\;$$

**Figure 2:**
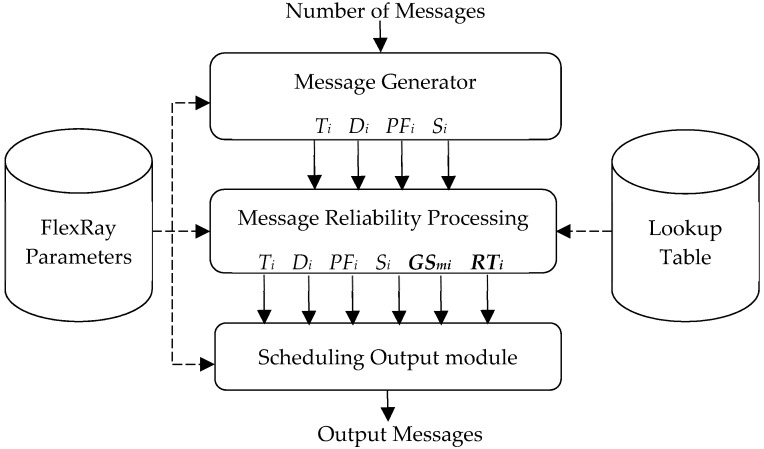
The proposed fast reliability scheduling algorithm (FRSA) architecture.

The proposed FRSA architecture and parameters according to the above retransmission mechanism are shown in Figure 2. It is composed of three units: the message generator and the message reliability processing and scheduling output modules. The FlexRay parameters and lookup table are some parameters required for the system and corresponding new *GS_mi_* values, respectively. In addition, there are two methods for the handling of a large number of messages in the proposed algorithm. First, each message is sorted by its *GS_mi_* in descending order; this step excludes the messages that do not need to be retransmitted. Second, in order to meet reliability and deadline constraints in the case of a greater number of messages, computation is performed using a lookup table method instead of an iterative solution. We describe the proposed algorithm in more detail in the next section.

### 2.2. Message Generator

A set of messages, *M*, is generated by the message generator module in the static segment. *M* = {*M*_1_, *M*_2_, …, *M_n_*}, and each message *M_i_* consists of four parameters, as in Equation (2), where *T_i_* denotes the generation rate of *M_i_*, and *D_i_* is the relative time of *M_i_* since it was produced until the end of transmission. *PF_i_* and *S_i_* are the probability of failure during transmission and the size of *M_i_* in bits, respectively. *PF_i_* can be computed as Equation (3), where the bit error rates (BER) value is decided according to the type of wire and the environment [15]. On the other hand, when the message is stored in a slot, its parameter *D_i_* should be less than or equal to *T_i_* to avoid being overwritten by other messages before transmission, as in Equation (4):

*M* = {*M_i_* (*T_i_*, *D_i_*, *PF_i_*, *S_i_*), *i* = 1, 2, …, *n*} (2)

(3) $$\;{PF}_{i}\;=\;1\;-\;{\left(1\;-\;\mathrm{BER}\right)}^{S_{i}}\;$$

(4) $$\;\forall \;m\;\in {M,\;D}_{i}\;\leq {\;T}_{i}\;$$

### 2.3. FlexRay Parameters

This module provides some parameters required of a message when the message *M*_i_ is transmitted over the static segment of FlexRay:

- Length of FlexRay communication cycle (*L_FC_*): the length of one communication cycle in FlexRay;
- Length of static segment (*L_ST_*): the static segment length of a FlexRay communication cycle;
- Number of static slots (*N_slot_*): total number of slots in the static segment, within the range 1–1023;
- Length of static slot (*L_slot_*): the length of a slot in the static segment, *L_slot_* = *L_ST_*/*N_slot_*;
- Testing time (*τ*): the time required to test fault tolerance in the system. *τ* is typically one hour (3,600,000 ms);
- System reliability goal (*SR*): reliability goal for the system. The reliability goal for a communication system (*SR*) means that the reliability of the system is necessary for transmitting a message.

### 2.4. Message Reliability Processing Module

**Figure 3:**
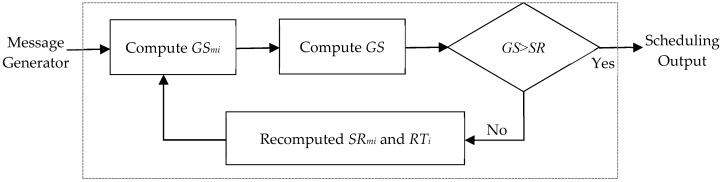
Flowchart of the message reliability processing module.

The FlexRay protocol is widely used for in-vehicle communication; however, it has no mechanism to prevent transient faults in its application layer. Therefore, improved communication reliability for FlexRay is important. The function of the message reliability processing module is to ensure reliability by computing the number of times a message delivery is duplicated for a static message in FlexRay. The flowchart of the message reliability processing module is shown in Figure 3. We attempt to increase the reliability of messages and then denote them with the following parameters:

- *GS_m__i_*: This estimates the probability of success (reliability) for a message (*M_i_*) in transmission, as in Equation (5), which is equal to that in [8]. *M_i_* consists of four parameters; in addition, it has a probability of failure and re-transmitted time (*RT_i_*). The parameter is used to improve the probability of success by re-transmission;
- Global success reliability (*GS*): This denotes the probability that all the messages (*M*) can be successfully transmitted at least once in the testing time (*τ*). If the reliability of message (*M*) which is computed in this module is bigger than or equal to the system reliability goal, the system is reliable in transmission and sends all messages to the scheduling output module. *M* consists of each message (*M_i_*); therefore, *GS* is obtained by multiplying each *GS_m__i_*, as in Equation (5):
  (5) $$\;GS=\overset{n}{\underset{i=1}{\prod }}{GS}_{mi}\;=\;\overset{n}{\underset{i=1}{\prod }}{{(1-PF}_{i}^{{RT}_{i}+1})}^{\frac{\tau }{T_{i}}}\;$$
- Single message reliability goal (*SR_mi_*): This parameter denotes the reliability goal of each message. *SR_mi_* must be bigger than or equal to the system reliability goal (*SR*). According to Equation (5), *GS* is obtained by multiplying each *GS_m__i_*: in fact, *GS* does not meet *SR* when the reliability goal of each message (*SR_mi_*) is equal to *SR*. This is because each *GS_m__i_* is less than 1 so that the multiplication result (*GS*) will fail to meet the system reliability goal.

A communication system is usually subject to reliability and deadline constraints (*D_i_*). The estimation of the failure probability of message transmission is critical for the scheduling algorithm. In this module, first, each message (*M_i_*) is sorted by *GS_mi_* in descending order. Sorting is a fast and effective method to reduce the procedure of the re-computed *SR_mi_* and *RT_i_*. For example, the order is *GS*_1_ > *GS*_4_ > *GS*_3_ > *GS*_2_ after sorting, if *GS*_1_ × *GS*_4_ > *SR,* and then these are multiplied by *GS*_3_. *SR_mi_* and *RT_i_* must be re-computed when the multiplication result fails to meet *SR*. The proposed method sorts each message (*M_i_*) and then excludes the messages that do not need to retransmitted to further improve slot utilization. How to obtain *SR_mi_* is described in the next section. 

### 2.5. Lookup Table Method

According to the above method, the FlexRay message *M* must be retransmitted to improve the communication reliability when *GS* fails to meet *SR*. The multiplication result of *GS_mi_* is defined as Equation (6), where *k* denotes the number of scheduled messages. We assume that *k* is 5 and *SR* is 0.99, which means *GS_mi_*^5^ ≥ 0.99, and *GM_mi_* for each scheduled message must be greater than or equal to 0.998. Then, by the square root formula, we can now convert Equation (6) into Equation (7), where *New_SR_mi_* is used to obtain a new *GS_mi_* value:

(6) $$G{S_{mi}}^{k}\;\geq \;\mathrm{System}\;\mathrm{Reliability}\;\mathrm{Goal}\;(SR)$$

(7) $$New\_SR_{mi}\;\geq \;\sqrt[{k}]{SR}\;$$

The communication requirements for automotive systems are the means of data stability and speed of processing. Many studies have been published on the method of calculating new *GS_mi_* values [8,10]. However, these methods have a high computational cost due to their iterative algorithm, and it is difficult to estimate the total running time of the procedure. In addition, the reliability of the global message may fail to meet the system reliability goal in the case of a greater number of messages. The proposed FRSA utilizes a pre-computed lookup table as a database to construct *New_SR_mi_* very quickly, where the *New_SR_mi_* is of a number of scheduled messages from 10 to 60. In general, the time complexity of the lookup table method and iterative algorithm are O(1) and O(n^2^), respectively, where n is related to the number of messages. This makes sure that the *New_SR_mi_* meets SR at only one step in time (O(1)) in this process. The proposed method improves the execution time over the previous algorithms, even in the case of a greater number of messages. This will be shown in the experimental results:

### 2.6. Scheduling Output Module

After the message reliability processing module, the next step will estimate the scheduling of all messages. The scheduling output module is used to make sure all messages can be transmitted before their deadline. Tanasa et al. [8] proposed bounding retransmissions to bound the minimum number of retransmissions required that must be done in order to achieve the system reliability goal. In the proposed method, this module is denoted with the following upper and lower bound constraint parameters:

- Maximum retransmission times (*RT_i_*_(max)_): slot utilization is also an important factor for successful transmission. When slot utilization is bigger than 1, it will cause message transmission failure. This means that the number of slots used for message retransmission is over the total number of slots in the static segment (*N_slot_*). Therefore, we can now denote *RT_i_*_(max)_ as Equation (8):
  (8) $$\overset{n}{\underset{i=1}{\sum }}({RT}_{i(\max)}+1)\;\leq {\;N}_{slot}\;$$
- Minimum retransmission times (*RT_i_*_(min)_): the proposed method improved the reliability of the system using a message re-transmission mechanism. Essentially, there are too few re-transmission times to achieve the system reliability goal (*SR*). *RT_i_*_(min)_ relates with *SR_mi_* and *PF_i_*, as in Equation (9):
  (9) $$\;{RT}_{i(\min)}\;\geq \;\frac{{\log(1-SR}_{mi}^{\frac{T_{i}}{\tau }})}{{\log PF}_{i}}\;-\;1\;$$

### 2.7. Proposed Fast Reliability Scheduling Algorithm

Algorithm 1 is the proposed fast reliability scheduling algorithm (FRSA). In the beginning, each message *M* is computed by the *RT_i_*_(min)_ and *GS_mi_* (lines 1 and 2). After obtaining these two values, we have the initial global success reliability (*GS*). 

**Algorithm 1** The proposed fast reliability scheduling algorithmInputs: *M*, System reliability goal (*SR*); Outputs: re-transmitted times (*RT_i_*) 1: *for* each *M_i_ do* 2:  compute *RT_i_*_(min)_ and *GS_mi_*  *end_for* 3: *if* (Global Success Reliability ≧ System Reliability goal) *then Schedulability Analysis*  /* GS does not meet SR, start to re-compute *SR_mi_* and *RT_i_*
*/ 4: *else sort GS_mi_* in descending order to obtain *GS*_1_ > *GS*_2_ >…> *GS_n_* (Equation (5))  *end_if* 5: *for j* = 1 *to n* − 1 *do* 6:  *until* ($\prod_{i=1}^{j}{{(1-PF}_{i}^{{RT}_{i}+1})}^{\frac{\tau }{T_{i}}}\geq SR$ &&$\;\prod_{i=1}^{j+1}{{(1-PF}_{i}^{{RT}_{i}+1})}^{\frac{\tau }{T_{i}}}\leq SR$) *then*   /* to exclude the messages that do not need to be re-transmitted */ 7:  *TempGS* = *SR*$/\prod_{i=1}^{j}{{(1-PF}_{i}^{{RT}_{i}+1})}^{\frac{\tau }{T_{i}}}$  *end_for* 8: *for I* = 1 *to n do* 9:   *if* (*GS_mi_* ≧ *TempGS*) *then RT_i_* = *RT_i_*_(min)_ 10:   *else* re-compute *RT_i_*_(min)_ and *GS_mi_* using *New*_*SR_mi_* =*TempGS*    *end_if* 11:   *if* (Global Success Reliability < System Reliability goal) 12:    *for i to n do* 13:     re-compute *RT_i_*_(min)_ and *GS_mi_* with *New*_*SR_mi_* using lookup table method     *end_for*    *end_if*  *end_for* 14: *if* (Global Success Reliability ≥ System Reliability goal) *then Schedulability Analysis* 15: *else goto* Line 4  *end_if* 16: *return* {*RT*_1_, *RT*_2_,…, *RT_n_*}

As in the discussion above on the characteristics of FlexRay, *GS* cannot meet the system reliability goal (*SR*) in most transmissions; therefore, *SR_mi_* and *RT_i_* are re-computed with recovery mechanism. Sorting is an important step, making the procedure of re-computation more effective (Line 4). To achieve an efficient execution time and slot utilization, the proposed algorithm multiplies each *GS_mi_* until the multiplication value does not meet *SR*, then the *tempGS* is used to obtain which messages have to be re-computed by the *SR_mi_* and *RT_i_* (Line 6 and 7). If the *GS_m__i_* is smaller than *tempGS*, the *New_SR_mi_* of the message is obtained from *tempGS* according to the *New_SR_mi_* (Line 10). In some cases, *GS* still does not meet *SR*, and the proposed mechanism re-computes the single message reliability goal for each message *M*, selected according to the lookup table method, which can provide a near-optional reliability goal for the reliability of the system (Line 13). Finally, we can check whether *GS* is bigger than or equal to the reliability goal or not (Line 14). If true, *schedulability analysis* processing is performed or re-computed by *SR_mi_* and *RT_i_* (Line 15). Based on algorithm analysis, the complexity of the proposed fast reliability scheduling algorithm is *O*(*n*^2^), where *n* is the number of messages.

## 3. Experimental Results

In this section, the experimental environment and validation for the proposed method are described as follows. Then, we will discuss the experimental results, comparing our approach and three related studies.

### 3.1. Experimental Environment and Validation

The proposed algorithm is implemented on a Windows 7 machine running an Intel(R) Core(TM) i7-4790 CPU @ 3.60GHz with visual studio 2015 by C++ language. The validation applied 51 cases which were generated for 10 to 60 messages: there are 100 examples in each case. The message parameters of FlexRay, such as *T_i_*, *D_i_*, *PF_i_*, and *S_i_*, are randomly generated. The periods and *PF_i_* are varied between 2 ms and 40 ms while the deadline is the same as the period and 1% and 50%, respectively. The period of a FlexRay communication cycle is 5 ms, *L_FC_* is 3 ms and *N_slot_* is 450, and *SR* is 0.99. The above parameter configurations are according to the BMW specification and are also adopted in the literature [8].

The validation of the proposed method uses three cases: message transmission with and without the proposed FRSA, with the other case unable to be scheduled. Data in Table 1 show the five messages which should be transmitted and their parameters. The global success reliability (*GS*) approaches 0 by applying Equation (5). *GS* does not meet *SR* because of being without a message re-transmission mechanism. In almost every case, the recovery mechanism is a necessary processing step to ensure the communication reliability of the FlexRay protocol. The test case applied the proposed FRSA to re-compute the *SR_mi_* and *RT_i_*; we can see that FRSA increases the *GS* to 0.9987. The proposed FRSA re-computed each corresponding *RT_i_*, and then messages *M*_1_~*M*_5_ are sorted by their *GS_mi_* in descending order. The overhead of the re-transmission mechanism is an increase of bandwidth utilization. The utilization increase of bandwidth (slot) can be explained because of the recovery mechanism for the re-transmitted messages.

There are two conditions which are unable to be scheduled. In one, the *GS* does not meet the system reliability goal (*SR*). In the other, the slot utilization is over the upper bound (*N*_slot_). This means that there is available bandwidth that can be used to re-transmit messages, meaning that the *RT_i_* rule cannot be followed, causing lower reliability. 

### 3.2. Performance Evaluation

In order to evaluate the performance of the proposed algorithm, we compared the execution time and slot utilization with existing algorithms [9,10,11]. This comparatively analyzes the related work to achieve the desired reliability target needed to determine the running time and slot usage. The slot utilization is related to the bandwidth utilization of FlexRay, as in Equation (10):

(10) $$\mathrm{Slot}\;\mathrm{Utilization}\;=\frac{used\;slots}{all\;slots}\;=\sum_{1}^{n}\frac{{RT}_{i}+1}{T_{i}}\text{×}\frac{L_{FC}}{{\;N}_{slot}}\;\leq \;1\;$$

These methods [9,10,11] show the execution time and slot utilization in experimental results; however, they did not discuss the relationship between performance (running time) and resource utilization (slot usage) when the number of messages is increased. Now that we have proposed a cost function, we can use it to analyze the performance and efficiency of previous works, as in Equations (11)–(13):

*cost* = *K*_1_ × *f*(∆*time*) + *K*_2_ × *f*(*slot*) (11)

*f*(∆*time*) = slope(*t_i_*, *t_i_*_−1_)/∆number of message × 100%, *i* = 2, …, *n* (12)

*f*(*slot*) = slot utilization/number of messages (13)

where *K*_1_ and *K*_2_ are the weight factors in the cost function, and they are the same and equal to 1 to balance the performance and resource utilization. *f*(∆*time*) is used to evaluate the rates of change (slopes) between the running time and the increased number of messages. Note that the *cost* value will be invalid in two cases: *f*(∆*time*) is negative when *t_i_* is smaller than *t_i_*_−1_, and the other is the lowest number of messages is the baseline for the slope function. *f*(*slot*) indicates the resource utilization of a single message in each experiment. 

First, the experimental data will be separated into three parts according to the different parameter configurations of existing methods. The message parameters are generated by random and each setting has 100 examples. The FlexRay bus parameters are fixed in Setting 1 and Setting 3, and are not fixed in Setting 2, as shown in Table 2. In addition, these results are also compared directly by *cost* value. The comparison and analysis of the proposed FRSA and existing methods in each setting are as follows.

• Comparison 1, Li’s method [9]

**Figure 4:**
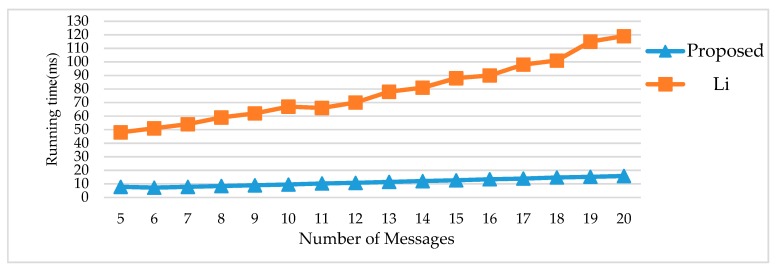
Comparison of running time with Li’s method.

In [9], there are 16 test cases with fixed parameters in FlexRay in the experiment. In particular, the probability of failure (*PF_i_*) is not related to the size of messages and the bit error rate, but is varied between 1% and 50%, respectively. The major focus of Li’s method is to save the bandwidth utilization rate. They defined an evaluation function to calculate the greatest contribution value, and then generated a new package of message re-transmission. This means that each message which is retransmitted makes a significant improvement in reliability and further improves slot usage. As the main aim of Li’s method, we can observe that work [9] has better performance in saving slot utilization than the proposed FRSA when the number of signals under 16, as Table 3 shows. However, along with an increase of the number of messages in test cases, the computational complexity of the evaluation function always causes a greater time cost than the proposed FRSA, as shown in Figure 4. When the performance evaluation is according to cost function, the fluctuating range of *f*(∆*time*) of Li’s method is more extreme than the proposed method. In addition, the average *cost* of the proposed method is better than Li’s method.

• Comparison 2, Wang’s method [11]

**Figure 5:**
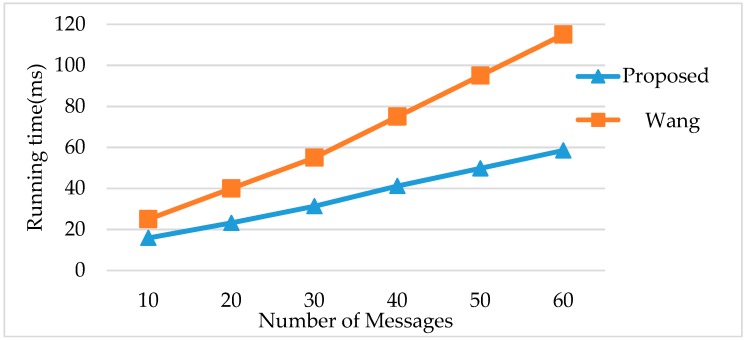
Comparison of running time with Wang’s method.

On the other hand, Wang et al. proposed a frame-packing algorithm based on transmission reliability (FPBTR) [11]. This algorithm is implemented by considering both reliable transmission and maximized bandwidth utilization. The comparison of the running time and slot utilization of the proposed FRSA algorithm and Wang’s method are shown in Table 4. According to the FPBTR method, the number of slots in the static segment (*N_slot_*) is dependent on the range of messages; they do not optimize static slot allocations. The proposed FRSA excluded messages which do not need to re-transmitted to further improve slot utilization. It can be found that the proposed FRSA has better performance in slot utilization than Wang’s method when the same parameters are utilized. Moreover, the FPBTR method has been focused on optimizing the frame packing of *L_slot_* and *N_slot_* which creates a greater time cost to meet the demands of the given reliability than the proposed FRSA, as shown in Figure 5.

• Comparison 3, Lee’s method [10]

**Figure 6:**
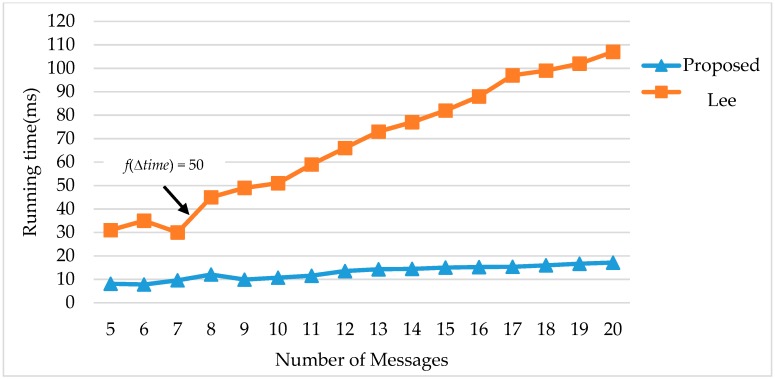
Comparison of running time with Lee’s method.

**Figure 7:**
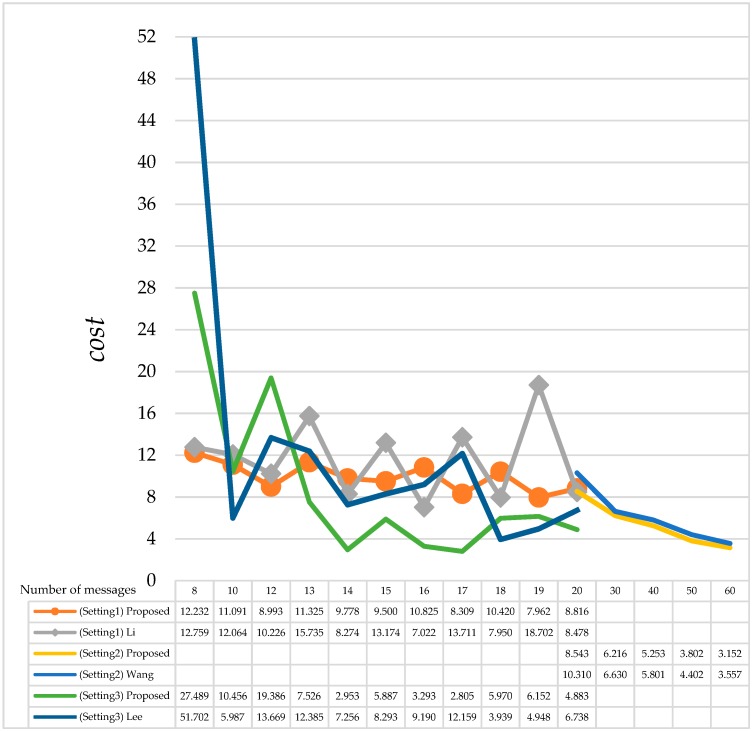
Comparison of *cost* with existing methods in different parameters.

Lee et al. [10] proposed a method to sort each message in descending order by its reliability value. After sorting, the new single message reliability goal is according to the reliability of the previous message. When the reliability goal does not satisfy reliability constraints, Lee’s method computes the average of the reliability value of messages; if the reliability of messages is smaller than the average value, then these messages should be corrected. The comparison of the slot utilization of the proposed FRSA algorithm and Lee’s method is shown in Table 5. Lee’s method has better performance in slot utilization in each test case; however, with the iterative method, it is difficult to estimate the execution time. Obviously, the *f*(∆*time*) of Lee’s method increased sharply when the number of messages is 8. These results suggest that its high computational complexities make the execution time increase more than the proposed FRSA, as shown in Figure 6. The average *cost* of the proposed FRSA is also better than Lee’s method, as shown in Figure 7.

After comparing the proposed method and the given methods [9,10,11], we summarize the main conclusions of our comparisons as shown in Table 6 and Table 7. We can see that the proposed FRSA method reduces execution time by an average of 70.76% because the computational complexity determines the performance of the algorithm. These results suggest that the proposed FRSA can achieve the goal of reliability more quickly by excluding parts of messages and the lookup table method than by the other iterative methods. On the other hand, we proposed a cost function to evaluate the performance and resources when the number of messages increases, as shown in Figure 7. Obviously, the total *cost* of the proposed FRSA is better than Wang’s method [11]. Although bandwidth utilization is not the best in each setting, the proposed FRSA still reduces on *cost* by an average of 13.33%.

## 4. Conclusions

Communication requirements for automotive systems include both data stability and speed of processing. Related vehicle applications such as powertrain systems, electronic stability control (ESC) and traction control systems (TCS) have response time constraints. This work presents a new fast reliability scheduling algorithm (FRSA) to recover the transient errors for the static segment of the FlexRay bus. The proposed method increased the reliability of the system by the re-transmission of messages to ensure communication requirements. Experimental results show that the proposed FRSA has better performance for the running time of finding optimal solutions than the existing methods. In addition, the proposed fast and low-time-complexity lookup table method makes not only a significant improvement on execution time, but is also suitable for any periodic time-triggered communication system.

## Figures and Tables

**Table 1:** The validation of the proposed method.

Recovery Mechanism	*M_i_* (*T_i_*, *D_i_*, *S_i_*, *PF_i_*)	*RT_i_*	*GS_mi_*	*GS*	Slot Utilization
Non	*M*_1_ (32, 32, 240, 2.39997 × 10^−5^)	0	0.0672055	0.0000	0.031424
	*M*_2_ (18, 18, 272, 2.71996 × 10^−5^)	0	0.00433948		
	*M*_3_ (24, 24, 296, 2.95996 × 10^−5^)	0	0.0117959		
	*M*_4_ (3, 3, 264, 2.63997 × 10^−5^)	0	1.74401 × 10^−14^		
	*M*_5_ (6, 6, 152, 1.51999 × 10^−5^)	0	0.000109455		
Proposed FRSA	*M*_1_ (32, 32, 240, 2.39997 × 10^−5^)	1	0.999935	0.9987	0.0628472
	*M*_3_ (24, 24, 296, 2.95996 × 10^−5^)	1	0.999869		
	*M*_5_ (6, 6, 152, 1.51999 × 10^−5^)	1	0.999861		
	*M*_2_ (18, 18, 272, 2.71996 × 10^−5^)	1	0.999852		
	*M*_4_ (3, 3, 264, 2.63997 × 10^−5^)	1	0.999164		

**Table 2:** The parameter settings of related work.

Message Parameter Configuration in Static Segments			
Parameter	Setting 1	Setting 2	Setting 3
Period (*T_i_*)	5 ms~20 ms	1 ms~25 ms	2 ms~40 ms
Deadline (*D_i_*)	same as *T_i_*	same as *T_i_*	same as *T_i_*
Size of message (*S_i_*)	64 bits	32 bits~72 bits	32 bits
Bit Error Rate (BER)	-	10^−3^	-
Probability of failure (*PF_i_*)	1%~50%	$1\;-\;{\left(1\;-\;\mathrm{BER}\right)}^{S_{i}}$	1%~50%
**The Parameter Configuration of FlexRay Buses**			
FlexRay cycle (*L_FC_*)	5 ms	5 ms	5 ms
Length of static segment (*L_ST_*)	3 ms	3 ms	3 ms
Length of slot in SS (*L_slot_*)	20 μs	8.3 μs~50 μs	8.57 μs
The number of slot in static segment (*N_slot_*)	150	60~360	350
**The Parameter Configuration of Systems**			
Range of messages	5~20	1~60	5~20
System reliability goal (*SR*)	99%	99%	99%
Testing time (τ)	3,600,000 ms	3,600,000 ms	3,600,000 ms

**Table 3:** Comparison of running time and slot utilization with Li’s method.

Number of Messages	Proposed Method		Li’s Method [9]	
	Running Time	Slot Utilization	Running Time	Slot Utilization
5	7.79	16.54	48	12
6	7.17	26.84	51	20
7	7.79	30.84	54	26
8	8.39	36.24	59	28
9	8.93	40.25	62	36
10	9.51	45.97	67	40
11	10.26	49.75	66	46
12	10.72	54.12	70	50
13	11.44	59.92	78	56
14	12.03	64.7	81	62
15	12.61	70.19	88	68
16	13.39	74.24	90	76
17	13.89	77.78	98	82
18	14.69	83.90	101	88
19	15.17	89.21	115	92
20	15.79	94.59	119	100

**Table 4:** Comparison of running time and slot utilization with Wang’s method.

Number of Messages	Proposed Method		Wang’s Method [11]	
	Running Time	Slot Utilization	Running Time	Slot Utilization
10	15.85	77.64	25	86
20	23.21	78.01	40	86.2
30	31.31	81.83	55	86.4
40	41.17	84.17	75	86.6
50	49.79	85.46	95	86.8
60	58.53	83.84	115	87.15

**Table 5:** Comparison of running time and slot utilization with Lee’s method.

Number of Messages	Proposed Method		Lee’s Method [10]	
	Running Time	Slot Utilization	Running Time	Slot Utilization
5	8.13	9.53	31	8.81
6	7.79	11.87	35	10.36
7	9.59	13.90	30	12.31
8	12.05	14.71	45	13.62
9	9.89	16.06	49	15.68
10	10.73	19.63	51	19.06
11	11.54	20.71	59	19.22
12	13.54	24.67	66	21.67
13	14.3	24.89	73	23.13
14	14.45	26.68	77	24.88
15	15.03	28.12	82	27.01
16	15.23	31.42	88	29.98
17	15.36	33.2	97	32.85
18	15.98	34.82	99	33.82
19	16.65	37.25	102	36.46
20	17.14	38.83	107	36.74

**Table 6:** Comparison of average running time with related studies.

	Methods	Average Running Time (ms)		Reduction (%)
Parameter		Proposed FRSA	Comparison Method	
**Setting 1**		11.22	77.94 (Li [9])	85.59
**Setting 2**		36.64	67.50 (Wang [11])	45.71
**Setting 3**		12.96	68.18 (Lee [10])	80.98
Average				70.76

**Table 7:** Comparison of average *cost* with related studies.

	Methods	Average *cost*		Reduction (%)
Parameter		Proposed FRSA	Comparison Method	
**Setting 1**		10.401	11.168 (Li [9])	6.86
**Setting 2**		5.393	6.140 (Wang [11])	12.16
**Setting 3**		10.101	12.782 (Lee [10])	20.97
Average				13.33
